# Serum and Urine Interferon Gamma-Induced Protein 10 (IP-10) Levels in Lupus Nephritis

**DOI:** 10.3390/jcm11113199

**Published:** 2022-06-03

**Authors:** Makayla P. Brady, Saiteja Chava, Shweta Tandon, Madhavi J. Rane, Michelle T. Barati, Dawn J. Caster, David W. Powell

**Affiliations:** 1 Department of Biochemistry and Molecular Genetics, School of Medicine, University of Louisville, Louisville, KY 40202, USA; makayla.brady@louisville.edu; 2 Department of Medicine, School of Medicine, University of Louisville, Louisville, KY 40202, USA; saiteja.chava@louisville.edu (S.C.); shweta.tandon@louisville.edu (S.T.); madhavi.rane@louisville.edu (M.J.R.); michelle.barati@louisville.edu (M.T.B.); david.powell@louisville.edu (D.W.P.)

**Keywords:** systemic lupus erythematosus, lupus nephritis, urinary biomarkers, serum biomarkers, IP10, interferon

## Abstract

Background: Lupus nephritis (LN) is a prevalent and severe complication of systemic lupus erythematosus (SLE). Non-invasive diagnostics are limited, and current therapies have inadequate response rates. Expression of the chemokine Interferon-γ-induced protein 10 (IP-10) is regulated by Interferon-γ signaling and NF-κB, and its molecular activity and enhanced urine concentrations are implicated in LN, but its utility as a diagnostic marker and association with demographic, clinical, or pathologic features is not defined. Methods: 38 LN patients and 11 patients with non-LN glomerular diseases (GD) with active disease were included. Eighteen of the LN patients had achieved remission at one follow-up during the study time. Serum and urine were obtained from these samples, and the IP-10 levels were measured. Results: Serum and urine IP-10 levels are significantly enhanced in LN patients with active disease as compared with normal individuals (serum average 179.7 pg/mL vs. 7.2 pg/mL, *p* < 0.0001; urine average 28.7 pg/mg vs. 1.6 pg/mg, *p* = 0.0019) and patients with other forms of glomerular disease (serum average 179.7 pg/mL vs. 84.9 pg/mL, *p* = 0.0176; urine average 28.7 pg/mg vs. 0.18 pg/mg, *p* = 0.0011). Urine IP-10 levels are significantly higher in patients with proliferative LN (PLN) than those with membranous LN (MLN) (average 32.8 pg/mg vs. 7.6 pg/mg, *p* = 0.0155). Urine IP-10 levels are also higher in MLN versus primary membranous nephropathy (MN) (average 7.6 pg/mg vs. 0.2 pg/mg, *p* = 0.0193). Importantly, serum IP-10 levels remain elevated during active LN and LN remission, but urine IP-10 levels are decreased from active LN to remission in 72% of our patients. Lastly, serum, but not urine IP-10 levels are significantly higher in African American than White American LN patients in active LN (average 227.8 pg/mL vs. 103.4 pg/mL, *p* = 0.0309) and during LN remission (average 254.6 pg/mL vs. 89.2 pg/mL, *p* = 0.0399). Conclusions: Our findings suggest that serum and urine IP-10 measurements provide promising tests for monitoring LN activity, differentiation between classifications of LN, and differentiation between LN and other forms of glomerular disease. We also conclude that further assessment of elevated IP-10 levels in the serum and urine of high-risk populations (i.e., African American) could be beneficial in determining why many of these patients have worse outcomes and are non-responsive to standard therapeutics.

## 1. Introduction

Systemic lupus erythematosus (SLE) is a complex autoimmune disease with genetic and immune-regulatory risk factors [1]. Kidney inflammation and injury, termed lupus nephritis (LN), occurs in about half of SLE patients, and is a leading cause of disability and death [2]. Achieving complete clinical remission strongly correlates with long-term kidney survival, but the rate of remission is only 40–60% at 2 years [2]. As many as 43% of patients with class IV LN, and 20% with class V LN, will go on to develop end-stage kidney disease [3]. Thus, new therapies with less toxicity and improved efficacy and noninvasive diagnostics to facilitate better disease management are drastically needed.

The chemokine CXCL10/interferon-γ-inducible protein-10 (IP-10) expression is regulated by interferon-γ signaling and NF-κB activity, and enhanced expression and activity is implicated in a number of renal diseases (mesangial proliferative glomerulonephritis, acute kidney injury, and LN) and transplant rejection [4,5,6]. T cells, neutrophils, monocytes, and endothelial cells secrete IP-10, and enhanced serum levels are reported in a number of systemic autoimmune diseases, including rheumatoid arthritis, systemic sclerosis, and SLE [7,8]. A role for IP-10 in LN is implicated from several reports showing enhanced IP-10 mRNA or protein levels in serum, urine, kidney biopsies, or circulating immune cells in patients with SLE with active LN [9,10,11,12,13]. A recent meta-analysis of studies investigating IP-10 as a potential biomarker for SLE/LN concluded that urinary, but not serum levels of IP-10 were significantly elevated in patients with active-LN versus active-SLE/without LN [14]. A possible role for IP-10 in the development of LN was also supported by the amelioration of nephritis in lupus-prone MRL/lpr mice with IP-10 receptor (CXCR3) deficiency [15].

The objectives of these studies were to evaluate IP-10 as a biomarker specific to LN, and to determine if the levels of IP-10 can be used to identify disease activity. The reported findings support a hypothesis that LN disease activity is associated with enhanced urine IP-10 levels, and that 1P-10 is, therefore, a viable candidate diagnostic marker for LN. We tested this hypothesis by evaluating the association of serum and urine IP-10 concentrations with disease parameters in LN patients, and with a comparison of levels in healthy individuals and patients with other glomerular diseases.

## 2. Experimental Section

### 2.1. Human Studies

The University of Louisville Internal Review Board approved all patient and normal individual sample collections and studies (IRB # 01.0536, continuation review approved 17 September 2021; IRB #96.0191, continuation review approved 4 May2022). All LN subjects had biopsy-proven LN and positive anti-nuclear (ANA) and/or anti-double stranded DNA (anti-dsDNA) antibodies at diagnosis. Disease status was determined through proteinuria levels measured on the day of collection. Patients with a proteinuria level >500 mg/g at the time of collection were considered to be in active LN. Non-lupus glomerular disease (GD) samples were considered to be active disease if proteinuria >1000 mg/g for IgAN, and >3500 mg/g for FSGS and MN. All urine IP-10 measurements were normalized to the urine creatinine levels measured on the sample date. Urine and serum samples were pulled for the collection dates that indicated active disease or remission. Due to the availability of the samples, the period of time between active and remission samples varied between patients. Some patient remission samples preceded their active date (flare), and some patient samples started the study in an active state and have since reached remission. The number of patients used in each ELISA analysis also varies, as some patients did not have urine and serum available from the same date. Approximately half of the patients never achieved complete remission; therefore, we were unable to use those patients in our comparison of active vs. remission samples. Healthy control donors had no underlying health conditions, were not on any medications, and were not anemic on screening. No other data for the control patients were available.

### 2.2. ELISA

Urine and serum from the same collection date were obtained from LN, phospholipase A2 receptor antibody (PLA2R) positive membranous nephropathy (MN), IgA nephropathy (IgAN), focal segmental glomerulosclerosis (FSGS) patients, and healthy control individuals. Two LN patients had urine, but did not have serum available on their active date, and 9 LN patients had serum, but did not have urine available on their active date. Levels of IP-10 in both urine and serum samples were determined by Human CXCL 10/IP10 Quantikine ELISA (R&D Systems, Minneapolis, MN, USA) following the manufacturer’s protocol. Samples were run undiluted in duplicate and triplicate.

### 2.3. Statistical Analysis

Statistics were run using the GraphPad Prism 8 software (San Diego, CA, USA). All statistical analyses are described in the figure legends. Experiments with 2 groups were analyzed using a Two-Tailed T-Test with Welch’s correction or a paired ratio T-Test. Experiments with 3 or more groups were analyzed using Brown–Forsythe and Welch’s ANOVA. For Figure 4, the data were tested using a nonparametric test (Independent Samples Mann–Whitney U test) using SPSS V28 (IBM corp, Armonk, NY, USA).

## 3. Results

### 3.1. Serum and Urine IP-10 Levels Are Enhanced in Patients with Active LN, and Urine IP-10 Levels Are Different for Patients with Proliferative versus Membranous LN

**Figure 1:**
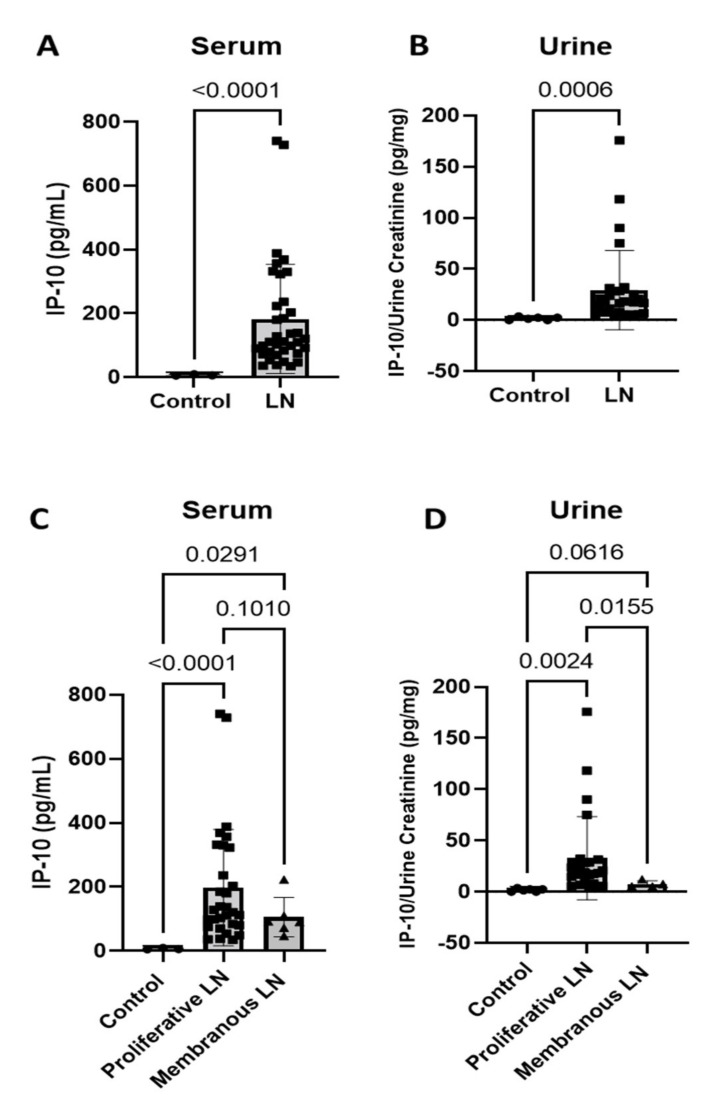
IP-10 is increased in serum and urine of LN patients. (**A**) Serum; and (**B**) urine levels of IP-10 of active LN samples are increased compared to healthy individuals (control) (serum, *n* = 3, *n* = 36; urine, *n* = 6, *n* = 29; Two-Tailed T-Test with Welch’s correction); (**C**) Serum; and (**D**) urine IP-10 levels in patients with active proliferative LN and membranous LN compared to control. In serum, both proliferative and membranous LN IP-10 levels were increased compared to control (serum, *n* = 3, *n* = 30, *n* = 6; Brown–Forsythe and Welch ANOVA Test, multiple comparisons). In urine, IP-10 levels were only increased in proliferative LN. (urine = 6, *n* = 25, *n* = 4; Brown–Forsythe and Welch ANOVA Test, multiple comparisons).

Clinical features and demographics of the 38 LN patients who had active disease used in this study are outlined in Table 1. IP-10 has been investigated as a possible serum or urine biomarker for LN in SLE patients, but results pertaining to IP-10 as a biomarker for activity or disease progression/remission are inconsistent, and biologic levels in other forms of glomerular diseases (GD) are unknown. Our active LN cohort had significant increases of IP-10 in both serum (Figure 1A) (*n* = 36; *p* < 0.0001) and urine (Figure 1B) (*n* = 29; *p* = 0.0006) compared to samples from normal individuals (serum *n* = 3, urine *n* = 6). Active LN was defined by a urine protein/creatinine ratio (UPCR) >500 mg/g. The LN cohort had an average UPCR of 2318 mg/g, and the GD cohort had an average UPCR of 6361 mg/g. Pathologic classification is a determinant of disease progression/outcome for LN [2,3]; these include class III (focal proliferative), class IV (diffuse proliferative), and class V (membranous (MLN)). Commonly, there are also patients with mixed proliferative and membranous lesions (III/V and IV/V). For our analyses, patients displaying class III, IV, or mixed lesions were included in the proliferative LN (PLN) group, and the MLN group had class V alone. Of the 38 patients included in the study, 32 patients displayed class III, IV, or mixed lesions, with an average UPCR of 2436 mg/g, and six patients displayed class V lesions alone, with an average UPCR of 3667 mg/g. Serum levels of IP-10 were significantly increased in PLN (*n* = 30; *p* < 0.0001) and MLN (*n* = 6; *p* = 0.0291) patients compared to healthy individuals (*n* = 3), and the PLN and MLN serum IP-10 levels were not significantly different (Figure 1C) (p = 0.1010). In contrast, urine IP-10 levels were significantly increased in PLN (*n* = 25) compared to MLN (*n* = 4; *p* = 0.0155) and control (*n* = 6; *p* = 0.0024), and MLN urine IP-10 levels were similar to those seen in healthy controls (Figure 1D) (*p* = 0.0616).

### 3.2. Urine IP-10 Levels Decrease with LN Remission

**Figure 2:**
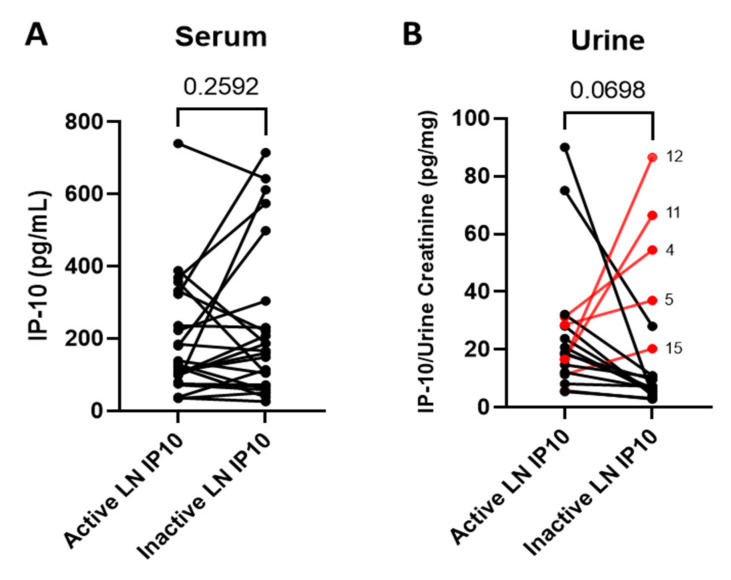
Urinary IP-10 decreases with remission in LN. (**A**) LN patient serum IP-10 levels from an active disease collection date compared to an inactive (remission) disease collection date; (**B**) urine levels of IP-10 of active and remission samples available from the same patient and collection dates as 2A (*n* = 18; Two-Tailed Ratio T-Test). Patients with an increase in urine IP-10 during remission are highlighted in red and labelled with the patient number that correlates to Table 2.

Changes in serum and urine IP-10 levels were assessed in patients that had both active and remission LN urine and serum samples available. Samples were collected during routine clinic follow-up, and the time between active and remission sample varied from a few months to a few years (range, 91–1728 days). Some subjects never achieved remission during the period observed. Out of the 38 patients included in this study, 18 patients had active and remission samples available for analysis. Active LN patient samples had an average UPCR of 2318 mg/g (low = 783 mg/g, high = 12681 mg/g), and samples collected on inactive/remission dates had an average UPCR of 226 mg/g (low = 108 mg/g, high = 366 mg/g). IP-10 levels showed no significant change in serum when patients went from an active LN state into remission (Figure 2A) (*n* = 18; *p* = 0.2592). IP-10 levels were not significantly decreased in urine when patients went from an active LN state into remission (Figure 2B) (*n* = 18; *p* = 0.0698); however, 72% (*n* = 13) of these patients had a decrease in urine IP-10 levels when they went from an active state into remission. Highlighted in red on Figure 2B are the patients that had an increase in urinary IP-10 during LN remission, as defined by proteinuria <500 mg/g. This accounted for 5 of the 18 patients (28%), and their points are labeled with the corresponding patient number outlined in Table 2. Table 2 shows the urine IP-10 levels and proteinuria levels for each of these patients in order of the highest active LN state urinary IP-10 level. This table also includes clinical characteristics from each patient at the time of collection. Of note, all five patients with increased IP-10 in remission had persistent elevations of anti-dsDNA, and two patients experienced increases in anti-dsDNA level. Patient 4 is of particular interest because complement levels were lower and anti-dsDNA levels were higher in the LN remission sample, suggesting active disease, despite a low level of proteinuria. Proteinuria increased at the following clinic visit, suggesting that the increase in IP-10 preceded the increase in proteinuria.

### 3.3. Differential Serum and Urine IP-10 Levels for LN versus Non-LN Glomerular Diseases

**Figure 3:**
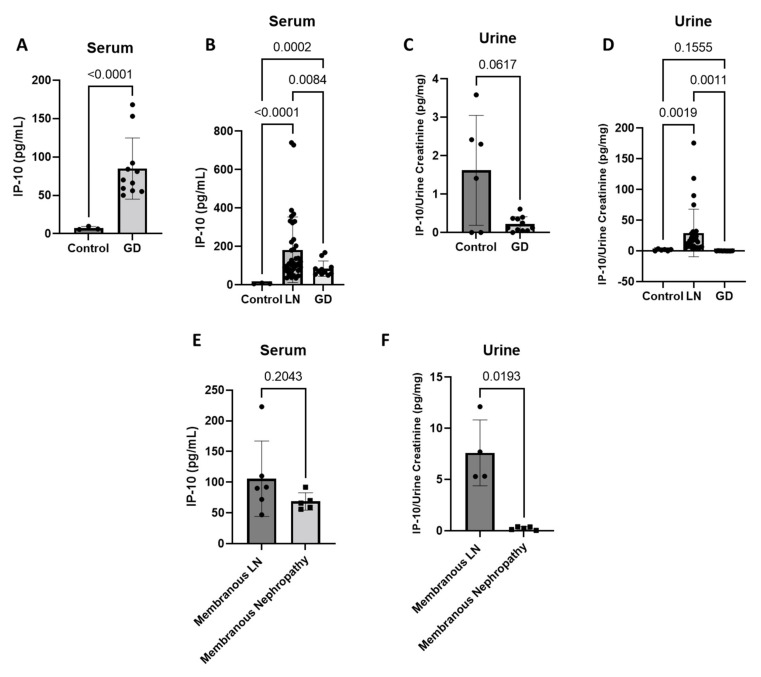
Urine IP-10 is increased in LN patients versus non-LN glomerular diseases (GD) and Membranous LN compared with primary Membranous Nephropathy. (**A**) GD serum compared to healthy individuals (control) (*n* = 3, *n* = 11; Two-Tailed T-Test with Welch’s correction); and (**B**) serum IP-10 is significantly increased in LN compared to the GD cohort, and both cohorts’ serum IP-10 levels are significantly increased compared to control (*n* = 3, *n* = 36, *n* = 11; Correction Brown–Forsythe and Welch ANOVA Test, multiple comparisons); (**C**) IP-10 urine levels are not significantly different in GD compared to control (*n* = 6, *n* = 11; Two-Tailed T-Test with Welch’s correction); and (**D**) in urine, IP-10 is significantly increased in LN compared to GD and control where GD is similar to control levels (*n* = 6, *n* = 29, *n* = 11; correction, Brown-Forsythe and Welch ANOVA Test, multiple comparisons); (**E**) patients with pure Membranous LN have similar serum IP-10 levels compared to patients with primary Membranous Nephropathy (*n* = 6, *n* = 5; Two-Tailed T-Test with Welch’s correction); and (**F**) patients with pure Membranous LN have increased urine IP-10 levels compared to patients with primary Membranous Nephropathy (*n* = 4, *n* = 5; Two-Tailed T-Test with Welch’s correction).

Serum and urine levels in samples collected during active LN were compared to samples collected from patients with active non-LN glomerular diseases (GD), including primary membranous nephropathy (MN), IgA nephropathy (IgAN), and focal segmental glomerulosclerosis (FSGS). All primary MN patients had phospholipase A2 receptor (PLA2R) antibody-mediated disease. Patients with non-LN GD displayed significant increases in serum IP-10 (Figure 3A) (*n* = 11) as compared with healthy individuals (*n* = 3) (controls) (*p* < 0.0001). LN patients (*n* = 36) had higher levels of serum IP-10 compared to patients with other forms of GD (*n* = 11; *p* = 0.0084), and both cohorts of patients had higher levels compared to the controls (*n* = 3) (Figure 3B) (control vs. LN, *p* < 0.0001; control vs. GD, *p* = 0.0002). Conversely, urine IP-10 for the non-LN GD patients (*n* = 11) was not significantly different than controls (Figure 3C) (*n* = 6; *p* = 0.0617). Urine IP-10 levels of LN patients (*n* = 29) was significantly higher than the urine IP-10 levels of non-LN GD patients (*n* = 11) and healthy control individuals (*n* = 6) (Figure 3D) (LN vs. GD, *p* = 0.0011; LN vs. control, *p* = 0.0019). Of the individuals with non-LN GD, 45% had primary MN (*n* = 5). These patients’ IP-10 levels were then compared to LN patients with pure class V MLN (serum *n* = 6, urine *n* = 4). Serum IP-10 levels between these two groups were similar (Figure 3E) (*p* = 0.2043); however, urine IP-10 levels in MLN were significantly higher than urine IP-10 levels in primary MN patients (Figure 3F) (*p* = 0.0193).

### 3.4. Differential Serum and Urine IP-10 Levels for African American versus White American LN Patients

**Figure 4:**
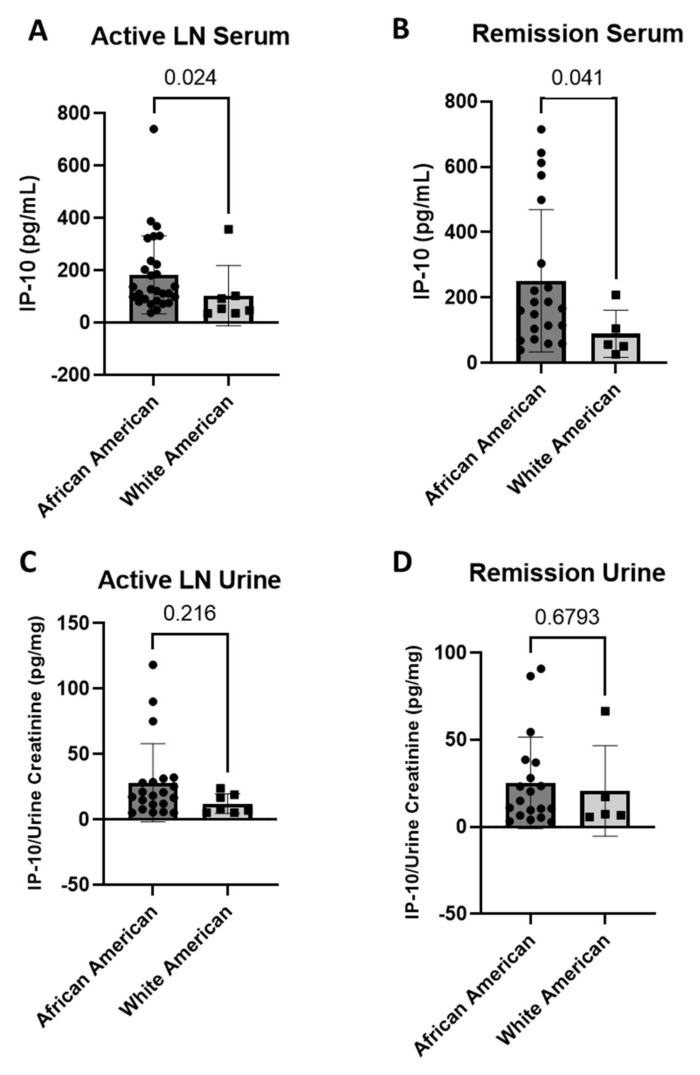
Serum and urine levels are different between African and white American LN patients. During inactive disease, (**A**) serum levels of IP-10 are significantly increased in African American patients compared to white American patients (*n* = 28, *n* = 7; Independent Samples Mann–Whitney U test); (**B**) serum IP-10 levels are also significantly increased in African American patients during remission compared to white American patients (*n* = 21, *n* = 5; Independent Samples Mann–Whitney U test); (**C**) urine IP-10 levels are not significantly different in African American compared to white American patients during active disease (*n* = 21, *n* = 7; Independent Samples Mann–Whitney U test); (**D**) there is also no significant difference of urine IP-10 during remission of the African American patients compared to white American patients (*n* = 19, *n* = 5; Independent Samples Mann–Whitney U test).

LN is known to have racial disparities where African American patients are more likely to develop severe manifestations of disease and respond poorly to the available treatment [16]. Our LN cohort primarily consisted of African American patients (76%, Table 1). Serum IP-10 levels are significantly increased during active LN (Figure 4A) (*p* = 0.024) and during LN remission (Figure 4B) (*p* = 0.041) in the African American patient group (active *n* = 29, remission *n* = 21) compared to white American patients (active *n* = 7, remission *n* = 5). Conversely, urine IP-10 levels are not significantly different between these groups in active LN (Figure 4C) (African American *n* = 21, white American *n* = 7; *p* = 0.216), or during LN remission (Figure 4D) (African American *n* = 19, white American *n* = 5; *p* = 0.6793).

## 4. Discussion

Defining improved noninvasive diagnostics and novel therapeutic targets for lupus nephritis (LN) will facilitate better disease management, and studies have implicated interferon (IFN)-γ-induced protein 10 (IP-10) as a candidate serum and urine marker for autoimmune activity and LN in SLE patients [9,10,11,12,13]. A meta-analysis published in 2019 thoroughly analyzed and reviewed data from 15 reports presenting serum and/or urine IP-10 protein levels in the context of disease activity in SLE patients [14]. These reports included a total of almost 2000 SLE patients and 2500 normal controls in mostly Asian, but a few European and North American cohorts, and three reports measured IP-10 in serum and urine, 11 reports measured IP-10 in serum only, and one report measured IP-10 in urine only, and only six reports focused on LN. Serum IP-10 was significantly elevated in active SLE compared to non-active SLE patients and healthy controls, but serum IP-10 levels were not different between patients with active and inactive LN. From a limited number of studies analyzing urine, IP-10 levels in urine tended to be higher in patients with active versus inactive LN, but did not reach statistical significance. A recent report by Ruacho et al., which was not included in the meta-analysis, investigated serum and urine levels of a panel of inflammatory proteins, including IP-10 in a Swedish cohort of 84 SLE patients, and 21 healthy controls [17]. All SLE patients were divided into low or high SLE disease activity groups, and serum and urine IP-10 was significantly higher in SLE versus healthy controls, and there was a correlation of urine and serum IP-10 levels with SLE disease activity. Patients were also divided into three LN status groups: never diagnosed, previously diagnosed but inactive, and active LN. Urine and serum IP-10 levels were significantly increased in the active versus inactive LN groups, but only urine IP-10 levels were significantly higher in the active LN versus the SLE without LN group.

With IP-10 serum and urine levels established in SLE/LN activity and healthy controls in these reports, the objectives of our LN-focused studies were to determine if IP-10 levels decrease with LN remission from patient follow-up, correlate with specific LN pathology, are different between LN and non-LN forms of GD, and to evaluate if there are differences between White and African American patients. Our study has several limitations, including small sample sizes, single center cohort, differences in time between active LN and remission samples, variable disease duration, and race/ethnicity/age/sex differences between LN and non-LN GD.

Consistent with other reports, we show that serum and urine IP-10 levels are enhanced in patients with active LN versus healthy controls. We also found that urine, but not serum IP-10 levels were decreased during LN remission compared with active LN for the majority of patients. A small number of patients had an increase of urine IP-10 in the setting of LN remission. For these patients, urine IP-10 may represent ongoing kidney inflammation. One patient with increased IP-10 during LN remission had a documented LN flare at the following clinic visit, suggesting that the increased remission urine IP-10 level may have indicated the impending LN flare. Recent studies have highlighted the discordance of proteinuria and biopsy findings in LN patients [18,19]. Novel biomarkers that can predict inflammation at the tissue level are needed.

When comparing White and African American patients with regards to active LN or remission, serum, but not urine IP-10 levels were significantly higher in African Americans during active disease and remission. Importantly, we also show that serum and urine IP-10 levels are higher in LN compared to non-LN forms of glomerular disease, suggesting that this could provide another differential test for LN. Interestingly, serum, but not urine levels of IP-10 were higher in non-LN forms of GD than healthy controls. IFN-γ is of interest to this report, as it regulates the expression of IP-10, and a recent report integrating urine proteomics and kidney single-cell genomics indicates that IFN-γ expression and its signaling axis are prominent in LN [20]. The proteomic analyses found that there was a urinary immune regulatory protein expression pattern that was shared by many patients with PLN, and the top function of these proteins is immune cell chemotaxis and cellular response to IFN-γ. A similar gene expression pattern was found in immune cells isolated from LN patient biopsies, suggesting that urine protein levels are indicative of the kidney immune activity in LN. A group also showed that urine levels of mRNA for IP-10, IP-10 receptor (CXCR3), TGF-β, and VEGF could accurately identify active class IV PLN, and that urine levels of these mRNAs were significantly reduced in patients who responded to therapy [21]. IFN-γ also regulates expression of CXCR3, TGF-β, and VEGF [22,23]. Another study showed that tubulointerstitial IP-10 mRNA expression decreased when the LN pathology change from proliferative class III or IV or mixed (class III/V or IV/V) to pure class V (MLN) [13]. Consistent with these findings and premise, we also found that urine levels of IP-10 protein were higher in PLN than in MLN patients in our cohort. We also show that urine IP-10 is significantly higher in MLN than in patients with primary MN. Accepted differential markers for primary MN are serum positivity for antibodies against PLA2R and glomerular expression of PLA2R protein, but some patients with MLN are PLA2R-positive [24,25]. Thus, collectively, these findings suggest that urine measurement of IP-10 and perhaps other IFN-γ-induced proteins should be considered as additional diagnostic tests for LN activity and as differential markers for PLN and MLN versus primary MN.

In addition to the diagnostic potential of urine and serum IP-10 testing, our findings presented in this report and those by others suggest that neutralizing IP-10 and other IFN proteins could provide therapeutic benefits to LN patients. IP-10 is expressed in higher levels in the colonic tissue and plasma of patients with ulcerative colitis (UC) [26]. Two phase II randomized studies were conducted to assess the efficacy and safety of a fully human monoclonal antibody to IP-10 (BMS-936557) in the treatment of moderately-to-severely active UC [26,27]. The primary and secondary endpoints were not met, but higher doses were associated with increased clinical response and histological improvements, and treatment was determined to be safe. A promising second-generation human IP-10 monoclonal antibody (BMS-986184) has also been developed [28].

The IFN family of cytokines (α, β, ε, κ, τ, δ, ζ, ω, ν, γ, λ), their signaling protein activators, and their effector proteins are collectively known as interferon signature, and are described as key mediators of SLE and LN [29,30]. IFNs type I, II, and III have been characterized based on immunological function [31]. Type I IFNs include α, β, ε, κ, τ, δ, ζ, ω, and ν isoforms, and have key roles in inflammation, tumor cell recognition, and T cell responses [32,33]. IFN-α is secreted by dendritic cells in response to the loss of immune tolerance, and activates T-cell differentiation in SLE [33,34]. Anifrolumab, a monoclonal antibody to type I interferon receptor, recently became the first FDA-approved medication targeting the interferon pathway in SLE [35]. A phase 2 trial (NCT02547922) evaluating anifrolumab in LN failed to meet the primary endpoint, but showed numerical improvement, and anifrolumab is now being evaluated in a phase 3 study in LN (NCT05138133) [36]. IFN-γ is the only member of type II IFN, and plays important roles in innate and adaptive immunity via the activation of chemotactic cytokines (chemokines) [34,37]. Emapalumab is human monoclonal antibody against IFN-γ that is FDA-approved for the treatment of primary hemophagocytic lymphohistiocytosis (HLH), a rare genetic disorder resulting in the overproduction of cytokines by histocytes and lymphocytes [38].

In conclusion, our findings suggest that serum and urine IP-10 levels help differentiate between pathologic classes of LN and LN compared to other forms of glomerular disease. Additionally, urine IP-10 measurement may be a promising test for monitoring disease activity in LN, with the majority of patients experiencing a decrease in urine IP10 during remission. Additional studies with urine obtained at the time of kidney biopsy would help better-define the correlation of urine IP-10 with LN activity. Finally, clinical trials evaluating the efficacy of monoclonal antibodies against IP-10, IFN-γ, and other IFN proteins in LN patients may lead to new therapeutic options and personalized treatments for patients with high serum and urine levels of IP-10, in high-risk populations (i.e., African American), or those non-responsive to standard therapeutics.

## Figures and Tables

**Table 1:** Clinical demographics of LN and GD cohort.

**Age in years (range)**		LN Patients Average = 35.9 (20.5–67)		**UPCR**				
		GD Patients Average = 48.4 (19–73)		LN Patient	Lupus Class	Active UPCR (mg/g)	GD Patient	UPCR (mg/g)
**Sex (%)**	LN patients			1	III/V	1151	1	4940
		Female	33/38 (86.8)	2	III	2073	2	4901
		Male	5/38 (13.2)	3	IV	1173	3	9538
				4	IV	2250	4	7708
	GD Patients			5	III/V	783	5	4357
		Female	4/11 (36.4)	6	III/V	1307	6	7303
		Male	7/11 (63.6)	7	IV	1329	7	11,000
**Race (%)**	LN Patients			8	IV	1509	8	10,000
		African American	29/38 (76.3)	9	IV	1333	9	1171
		White	8/38 (21.1)	10	IV/V	2645	10	4483
		Asian	1/38 (2.6)	11	III/V	1859	11	4571
	GD Patients			12	IV/V	3507		
		African American	1/11 (9.1)	13	IV/V	1489		
		White	7/11 (63.6)	14	V	1439		
		Asian	2/11 (18.2)	15	III/V	905		
		Other	1/11 (9.1)	16	IV	1554		
				17	IV/V	2928		
**Anti DS DNA Positive**			27/37 (73.0)	18	IV/V	1712		
**Low C3/C4 (%)**			23/37 (62.3)	19	IV/V	4791		
**Lupus Class (%)**				20	IV	3633		
		III	3/38 (7.9)	21	IV/V	2354		
		IV	10/38 (26.3)	22	III	2679		
		V	6/38 (15.8)	23	III	923		
		III/V	8/3 (21.1)	24	V	1769		
		IV/V	11/38 (28.9)	25	IV	2746		
				26	V	12,681		
**Immunosuppression at collection of samples (%)**				27	V	1066		
Mycophenolate			33/38 (86.8)	28	IV/V	1768		
Prednisone			27/38 (71.1)	29	IV	1127		
Hydroxycholorquine			26/38 (68.4)	30	III/V	3553		
Belimumab			5/38 (13.2)	31	III/V	1355		
Methotrexate			1/38 (2.6)	32	III/V	1513		
Tacrolimus			4/38 (10.5)	33	IV	2427		
Cyclophosphamide			4/38 (10.5)	34	IV/V	6355		
Rituximab			4/38 (10.5)	35	IV/V	14,611		
				36	IV/V	928		
				37	V	3819		
				38	V	1229		

**Table 2:** IP-10 urine levels, proteinuria levels, and clinical characteristics from the same patients when they were in remission or in an active flare. This table corresponds with Figure 2B. Results highlighted in red indicate an abnormal result for that clinical test, with normal ranges described below the table. Not all patients had their complement and anti-dsDNA titer available, and those data are indicated as “NC” for not collected.

Sample	Active LN Urine IP10/Creatinine (pg/mg)	Inactive LN Urine IP10/Creatinine (pg/mg)	Active UPCR (mg/g)	Inactive UPCR (mg/g)	Active LN C3/C4 (mg/dL)	Active LN anti-dsDNA (IU/mL)	Inactive LN C3/C4 (mg/dL)	Inactive LN anti-dsDNA (IU/mL)	Time between Samples (Days)
1	90	4.225	1151	211	122/30	11	103/30	11	665
2	75	28.017	2073	108	54/3	2318	99/11	74	154
3	32.099	10.945	1173	119	68/16	350	94/14	184	133
4	31.25	54.472	2250	285	60/13	739	43/6 ^ᵻ^	6035 ^ᵻ^	294
5	28.571	36.986	783	301	112/30	16	107/26	15	91
6	28.125	4.124	1307	196	71/10	24	99/12	9	924
7	23.78	5.634	1329	254	130/19	50	107/14	81	378
8	20.755	5.357	1509	226	74/28	23	79/23	13	895
9	18.841	6.667	1333	333	89/20	37	93/21	43	721
10	18.28	9.589	2645	205	73/18	11	62/14	6	938
11	16.667	66.423	1859	117	106/11	181	143/18	34	826
12	16.592	86.567	3507	209	79/17	50	85/19	37	928
13	14.674	10.484	1489	258	92/10 *	55 *	57/13	34	1155
14	12.121	6.618	1439	125	171/52	1	145/34	1	525
15	11.385	20.283	905	170	93/37	8	99/33	19	1050
16	8.108	7.317	1554	366	81/15	7	79/11	6	1525
17	5.797	2.857	2928	362	NC	NC	104/26	116	1175
18	5.479	3.125	1712	164	136/38	14	NC	NC	1728

NC = not collected; ^ᵻ^ LN flare on following visit (1800 mg/g); * value not same day, but within 4 weeks of urine sample; Normal Ranges: C3 80-150mg/dL, C4 18-55mg/dL, anti-dsDNA ≤ 4IU/mL.

## Data Availability

The data presented in this study are available on request from the corresponding author.

## Abbreviations

LN (Lupus Nephritis), SLE (Systemic Lupus Erythematosus), IP-10 (Interferon γ-Induced Protein 10), IFN (Interferon), GD (Non-LN Glomerular Diseases), PLN (Proliferative LN), MLN (Membranous LN), MN (Membranous Nephropathy), IgAN (IgA Nephropathy), FSGS (Focal Segmental Glomerulosclerosis), UPCR (Urine Protein to Creatinine Ratio).

## References

[1] Tsokos G.C. (2011). Systemic lupus erythematosus. N. Engl. J. Med..

[2] Anders H.J., Saxena R., Zhao M.H., Parodis I., Salmon J.E., Mohan C. (2020). Lupus nephritis. Nat. Rev. Dis. Primers.

[3] Tektonidou M.G., Dasgupta A., Ward M.M. (2016). Risk of End-Stage Renal Disease in Patients with Lupus Nephritis, 1971–2015: A Systematic Review and Bayesian Meta-Analysis. Arthritis Rheumatol..

[4] Gao J., Wu L.L., Wang S.Y., Chen X.M. (2020). Role of Chemokine (C-X-C Motif) Ligand 10 (CXCL10) in Renal Diseases. Mediat. Inflamm..

[5] Groom J.R., Luster A.D. (2011). CXCR3 ligands: Redundant, collaborative and antagonistic functions. Immunol. Cell Biol..

[6] Engel M.A., Neurath M.F. (2010). Anticancer Properties of the IL-12 Family—Focus on Colorectal Cancer. Curr. Med. Chem..

[7] Antonelli A., Ferrari S.M., Giuggioli D., Ferrannini E., Ferri C., Fallahi P. (2014). Chemokine (C-X-C motif) ligand (CXCL)10 in autoimmune diseases. Autoimmun. Rev..

[8] Lee E.Y., Lee Z.H., Song Y.W. (2009). CXCL10 and autoimmune diseases. Autoimmun. Rev..

[9] El-Gohary A., Hegazy A., Abbas M., Kamel N., Nasef S.I. (2016). Serum and Urinary Interferon-Gamma-Inducible Protein 10 in Lupus Nephritis. J. Clin. Lab. Anal..

[10] Nasef S.I., El-Gohary A., Hegazy A., Abbas M.A.F., Kamel N. (2016). Serum and Urinary Interferon-Gamma-Inducible Protein 10 in Lupus Nephritis. Ann. Rheum. Dis..

[11] Abujam B., Cheekatla S., Aggarwal A. (2013). Urinary CXCL-10/IP-10 and MCP-1 as markers to assess activity of lupus nephritis. Lupus.

[12] Fu Q., Chen X.Q., Cui H.J., Guo Y.Z., Chen J., Shen N., Bao C.D. (2008). Association of elevated transcript levels of interferon-inducible chemokines with disease activity and organ damage in systemic lupus erythematosus patients. Arthritis Res. Ther..

[13] Lu J.X., Kwan B.C.H., Lai F.M.M., Choi P.C.L., Tam L.S., Li E.K.M., Chow K.M., Wang G., Li P.K.T., Szeto C.C. (2011). Gene expression of TWEAK/Fn14 and IP-10/CXCR3 in glomerulus and tubulointerstitium of patients with lupus nephritis. Nephrology.

[14] Puapatanakul P., Chansritrakul S., Susantitaphong P., Ueaphongsukkit T., Eiam-Ong S., Praditpornsilpa K., Kittanamongkolchai W., Avihingsanon Y. (2019). Interferon-Inducible Protein 10 and Disease Activity in Systemic Lupus Erythematosus and Lupus Nephritis: A Systematic Review and Meta-Analysis. Int. J. Mol. Sci..

[15] Steinmetz O.M., Turner J.E., Paust H.J., Lindner M., Peters A., Heiss K., Velden J., Hopfer H., Fehr S., Krieger T. (2009). CXCR3 mediates renal Th1 and Th17 immune response in murine lupus nephritis. J. Immunol..

[16] Lea J.P. (2002). Lupus nephritis in African Americans. Am. J. Med. Sci..

[17] Ruacho G., Lira-Junior R., Gunnarsson I., Svenungsson E., Bostrom E.A. (2022). Inflammatory markers in saliva and urine reflect disease activity in patients with systemic lupus erythematosus. Lupus Sci. Med..

[18] Malvar A., Alberton V., Lococo B., Ferrari M., Delgado P., Nagaraja H.N., Rovin B.H. (2020). Kidney biopsy–based management of maintenance immunosuppression is safe and may ameliorate flare rate in lupus nephritis. Kidney Int..

[19] De Rosa M., Azzato F., Toblli J.E., De Rosa G., Fuentes F., Nagaraja H.N., Nash R., Rovin B.H. (2018). A prospective observational cohort study highlights kidney biopsy findings of lupus nephritis patients in remission who flare following withdrawal of maintenance therapy. Kidney Int..

[20] Fava A., Buyon J., Mohan C., Zhang T., Belmont H.M., Izmirly P., Clancy R., Monroy-Trujillo J., Berthier C., Davidson A. (2022). Urine Proteomics and Single Cell Transcriptomics Identify IL-16 as a Biomarker for Lupus Nephritis. Arthritis Rheumatol..

[21] Avihingsanon Y., Phumesin P., Benjachat T., Akkasilpa S., Kittikowit V., Praditpornsilpa K., Wongpiyabavorn J., Eiam-Ong S., Hemachudha T., Tungsanga K. (2006). Measurement of urinary chemokine and growth factor messenger RNAs: A noninvasive monitoring in lupus nephritis. Kidney Int..

[22] Nakamura M., Manser T., Pearson G.D.N., Daley M.J., Gefter M.L. (1984). Effect of Ifn-Gamma on the Immune-Response Invivo and on Gene-Expression Invitro. Nature.

[23] Barrat F.J., Crow M.K., Ivashkiv L.B. (2019). Interferon target-gene expression and epigenomic signatures in health and disease. Nat. Immunol..

[24] Beck L.H., Bonegio R.G.B., Lambeau G., Beck D.M., Powell D.W., Cummins T.D., Klein J.B., Salant D.J. (2009). M-type phospholipase A2 receptor as target antigen in idiopathic membranous nephropathy. N. Engl. J. Med..

[25] Safar-Boueri L., Piya A., Beck L.H., Ayalon R. (2021). Membranous nephropathy: Diagnosis, treatment, and monitoring in the post-PLA2R era. Pediatr. Nephrol..

[26] Mayer L., Sandborn W.J., Stepanov Y., Geboes K., Hardi R., Yellin M., Tao X., Xu L.A., Salter-Cid L., Gujrathi S. (2014). Anti-IP-10 antibody (BMS-936557) for ulcerative colitis: A phase II randomised study. Gut.

[27] Sandborn W.J., Colombel J.F., Ghosh S., Sands B.E., Dryden G., Hebuterne X., Leong R.W., Bressler B., Ullman T., Lakatos P.L. (2016). Eldelumab [Anti-IP-10] Induction Therapy for Ulcerative Colitis: A Randomised, Placebo-Controlled, Phase 2b Study. J. Crohns Colitis.

[28] Cai W.G., Leil T.A., Gibiansky L., Krishna M., Zhang H.W., Gu H.D., Sun H.D., Throup J., Banerjee S., Girgis I. (2020). Modeling and Simulation of the Pharmacokinetics and Target Engagement of an Antagonist Monoclonal Antibody to Interferon-gamma-Induced Protein 10, BMS-986184, in Healthy Participants to Guide Therapeutic Dosing. Clin. Pharm. Drug Dev..

[29] Chyuan I.T., Tzeng H.T., Chen J.Y. (2019). Signaling Pathways of Type I and Type III Interferons and Targeted Therapies in Systemic Lupus Erythematosus. Cells.

[30] Ronnblom L., Leonard D. (2019). Interferon pathway in SLE: One key to unlocking the mystery of the disease. Lupus Sci. Med..

[31] Platanias L.C. (2005). Interferon signals: What is classical and what is nonclassical?. J. Interf. Cytok. Res..

[32] Jiang J., Zhao M., Chang C.S., Wu H.J., Lu Q.J. (2020). Type I Interferons in the Pathogenesis and Treatment of Autoimmune Diseases. Clin. Rev. Allergy Immunol..

[33] Sprooten J., Agostinis P., Garg A.D. (2019). Type I interferons and dendritic cells in cancer immunotherapy. Int. Rev. Cell Mol. Bio..

[34] Platanias L.C. (2005). Mechanisms of type-I- and type-II-interferon-mediated signalling. Nat. Rev. Immunol..

[35] Mullard A. (2021). FDA approves AstraZeneca’s anifrolumab for lupus. Nat. Rev. Drug Discov..

[36] Jayne D., Rovin B., Mysler E.F., Furie R.A., Houssiau F.A., Trasieva T., Knagenhjelm J., Schwetje E., Chia Y.L., Tummala R. (2022). Phase II randomised trial of type I interferon inhibitor anifrolumab in patients with active lupus nephritis. Ann. Rheum. Dis..

[37] Lee A.J., Ashkar A.A. (2018). The Dual Nature of Type I and Type II Interferons. Front. Immunol..

[38] Merli P., Algeri M., Gaspari S., Locatelli F. (2020). Novel Therapeutic Approaches to Familial HLH (Emapalumab in FHL). Front. Immunol..

